# (In)temperance among Women

**Published:** 1902-11-15

**Authors:** 


### (IN)TEMPERANCE AMONG WOMEN.

UNDER the somewhat deceptive heading of "Temperance among Women," the newspapers of last week chronicled the proceedings of a meeting held at the Church House, Westminster, under the presidency of Mrs. Creighton, for the purpose of discussing the question of "the present-day dangers of intemperance among women and the best methods of meeting them." The meeting was convened by the Women's Union of the Church of England Temperance Society; but the principal speakers were Sir Thomas Barlow and the Bishop of London, and, save the president herself, no woman is reported as having taken any part in the debate. Possibly their abstention may in some degree account for the entirely unpractical tone by which it seems to us to have been characterised.

It seems to be a matter of common sense that the amount of effort directed towards the suppression of any recognised evil should bear some definite proportion to the magnitude of the evil itself, and to the gravity of its effects upon the community. Notwithstanding this, Sir Thomas Barlow is reported to have said that "it was needless for him to dwell on the magnitude of the evils brought about by intemperance among women." It appears to us that nothing was more "needful"; because it is on this very question of magnitude that the related question of the measures to be adopted for its suppression should mainly turn. A drunken wife and mother is no doubt a very serious evil to her immediate environment; and she every now and then obtains a paragraph all to herself in the records of a police court, and is rendered for the time a public character. What numerical proportion does she really bear to the vast number of just women who either abstain from alcohol or take it only in strict moderation? One of the later speakers may perhaps be regarded as having made some attempt to solve this problem, for he is reported as having told his audience, but without saying on what authority, that there are now in London 8,900 women who have been convicted of drunkenness more than ten times. These might certainly be regarded as "habitual drunkards." The word "London" is a little vague; but, as the speaker was referring to convictions, we may assume that he meant the London of the Metropolitan Police district, which contains more than six millions and a half of people. Of these, according to ordinary proportions, nearly three and a half millions would be females, of whom about 1,370,000 would be over 30 years of age. Putting the matter in this way, it would appear that, in London, there may be a single drunkard to every 154 women over the age of 30, or one to every 386 of the female population. When the facts are thus regarded, we obtain an approximate measure of what female drunkenness means in relation to women generally; it being borne in mind, of course, that the temptations to and the causes of excess are much more prevalent among a crowded urban population than elsewhere, and that London is a place towards which a good deal of the general immorality of the country has a natural tendency to gravitate. We are inclined to think that the proportion of female drunkenness thus disclosed is hardly, if at all, in excess of the natural waste of such an aggregation of human atoms. The stress of life is too much for many of the feeble; and the solace which they seek from drink may often be the effect rather than the cause of their downfall.

We do not think that Sir Thomas Barlow materially advanced the discussion by his pronouncement that drunkenness "should be treated primarily and throughout as a sin." A sin, according to Johnson, is "an act against the laws of God, a violation of the laws of religion," and such acts and violations are not "treated" at all by the community, unless they also constitute offences against the law. Individuals may shun those whom they regard as sinners, or they may seek them out in the hope of promoting their reformation. Certain religious communities have required from "sinners" the performance of public penances; but the efficacy of these is at least doubtful, and their enforcement would no longer be aided by any considerable section of public opinion. It would have been more satisfactory if Sir Thomas had addressed himself to the scientific aspects of the question, and had told his audience in what degree drunkenness should be regarded as a disease, to what extent it is controllable by medical treatment, and how far the stability of the nervous system of a drunkard is likely to recover under the influence of total abstinence. These are the sort of data which philanthropists and the public may reasonably expect from the medical profession; and, when thus seeking bread, it is hard that they should be put off with a stone. But the climax of the Westminster meeting was reached by the Bishop of London, who, after deploring the prevalence of the morphia habit among women, especially in the West End of London, produced his panacea for the cure of the evil which he and his audience were assembled to deplore. His lordship declared that if we "were to give our working people better homes, and to see that they got a wage on which they could live and bring up their families, we should not have so much trouble with drunkenness." In other words, if we were to remove the stress of life, its worst consequences would no longer occur. Exactly so; and when the sky falls, we shall catch larks.

